# Temporal Variations in Soil Moisture for Three Typical Vegetation Types in Inner Mongolia, Northern China

**DOI:** 10.1371/journal.pone.0118964

**Published:** 2015-03-17

**Authors:** Hao Zheng, Jixi Gao, Yanguo Teng, Chaoyang Feng, Meirong Tian

**Affiliations:** 1 College of Water Sciences, Beijing Normal University, Beijing, 100875, China; 2 Nanjing Institute of Environmental Sciences, Ministry of Environmental Protection of China, Nanjing, Jiangsu Province, 210042, China; 3 Chinese Research Academy of Environmental Sciences, Beijing, 100012, China; Institute of Botany, CHINA

## Abstract

Drought and shortages of soil water are becoming extremely severe due to global climate change. A better understanding of the relationship between vegetation type and soil-moisture conditions is crucial for conserving soil water in forests and for maintaining a favorable hydrological balance in semiarid areas, such as the Saihanwula National Nature Reserve in Inner Mongolia, China. We investigated the temporal dynamics of soil moisture in this reserve to a depth of 40 cm under three types of vegetation during a period of rainwater recharge. Rainwater from most rainfalls recharged the soil water poorly below 40 cm, and the rainfall threshold for increasing the moisture content of surface soil for the three vegetations was in the order: artificial *Larix* spp. (AL) > *Quercus mongolica* (QM) > unused grassland (UG). QM had the highest mean soil moisture content (21.13%) during the monitoring period, followed by UG (16.52%) and AL (14.55%); and the lowest coefficient of variation (CV 9.6-12.5%), followed by UG (CV 10.9-18.7%) and AL (CV 13.9-21.0%). QM soil had a higher nutrient content and higher soil porosities, which were likely responsible for the higher ability of this cover to retain soil water. The relatively smaller QM trees were able to maintain soil moisture better in the study area.

## Introduction

Soil moisture in semiarid areas plays an important role in ecological hydrological processes, including evapotranspiration, infiltration, runoff, and erosion [1, 2]. Maintaining a high level of soil-moisture content (SMC) can improve the capacity of ecological systems to conserve water [3, 4]. Variations in soil moisture can be highly influenced by plant-soil interactions [5]. The type of vegetation is often associated with specific soil properties and different community structures and architectures, therefore variation in vegetation can be a major influence on the pattern of soil moisture [6]. In past decades, some research has suggested that soil and water conservation in ecosystems could be improved by increasing forest cover. More recent studies, however, have suggested that forests with high coverage may not be the best option for increasing SMC and water resources, especially in arid or semiarid areas [7, 8]. Determining the effect of different types of vegetation on SMC is thus very important for the management of soil-water conservation in forest watersheds.

Soil moisture in ecosystems has received increasing attention recently [9], and many authors have discussed the spatial and temporal characteristics of soil moisture [10–12]. Only a few studies, however, have taken into account the variability of soil moisture at different soil depth, and the characterization of temporal variability in soil moisture remains one of the challenges in the hydrological sciences [13, 14]. To further understand the migration and transformation of rainwater in soil, we have detailed the temporal variation of soil moisture for three vegetation covers to a depth of 40 cm at 15-min intervals.

The experiments were performed at the Saihanwula National Nature Reserve, which is located in a semiarid area of northern China. The reserve has fragile hydrological conditions that are very sensitive to changes in the vegetation cover. A portion of the vegetation dies or never matures due to drought and water shortages, which leads to a reduced ability of ecosystems to conserve water and ultimately to desertification. The effect of vegetation cover on soil moisture in this area of water shortage has not been studied through long-term and dynamic observations. The objectives of this study were thus to 1) monitor SMCs for three types of vegetation on a long-term and dynamic basis, 2) analyze the SMC variation to a depth of 40 cm, and 3) identify the relationships between SMC and various soil properties.

## Materials and Methods

### Study Area

The study was carried out in the Saihanwula National Nature Reserve, located in a semiarid area of northern China (43°59′ to 44°27′N and 118°18′ to 118°55′E). The administrative body of this reserve gave permission for our research activities. Our study area was not in the core region of the reserve, and our activities did not affect any endangered or protected species. The reserve contains the headwaters of the West Liao River and is in the transition zone between the broadleaved forests of eastern Asia and the coniferous forests of the Greater Hinggan Mountains and between grassland and forest. The climate of the study area is classified as temperate semiarid, with long cold winters and short hot summers. The average annual hours of sunshine and temperature are 3000 h and 2°C, respectively. The average annual rainfall is 400 mm, of which more than 80% falls during the rainy season. The major natural vegetation in the region includes *Larix* spp., *Betula platyphylla* Suk., *Quercus mongolica* F., *Populus davidiana*, and *Prunus sibirica*, as well as herbaceous plants such as *Stipa baicalensis* Roshev, *Artemisia sacrorum*, and *Filifolium sibiricum* L. The dominant soil types are mountain Phaeozem, Greyzems, Brunisolic soil, and chestnut soil.

### Data Collection

Data was collected on rainfall, soil moisture, and other soil properties. The automatically and manually measured parameters are presented in Table 1.

**Table 1:** Automatically and manually measured parameters.

Parameters	Definition	Measurement method	Measurement time
R (mm)	rainfall	HOBO rain gauges	2013.06.10 to 2013.09.29
SMC (%)	soil moisture content	EM 50	
T_soil_	soil temperature	EM 50	
ST (%)	soil texture	hydrometer	2013.09 to 2013.10
ρ_b_ (g/cm^3^)	soil bulk density	cutting ring method	
SP	soil porosity	cutting ring method	
pH	pH	pH meter	
SOM (g/kg)	soil organic matter	potassium dichromate capacity titration	
TN (g/kg)	total nitrogen	semi-micro Kjeldahl	
TP (g/kg)	total phosphorus	molybdenum blue	
TK (g/kg)	total potassium	flame photometry	

### Site selection

The experiments were performed on moderately sunny slopes in the reserve from June to September, 2013. Soil moisture was monitored at three vegetation sites (50 × 50 m): tall artificial *Larix* spp. trees (AL), smaller *Q*. *mongolica* trees (QM), and unused grassland (UG). The study area is at the northern and eastern limit in China for the growth of *Larix* spp., which is the most commonly planted tree in the reserve. *Q*. *mongolica* is widely distributed on sunny and semi-sunny slopes, in valleys, and in sandy soil, and occupies 12.59% of the area of the reserve. Grassland is the major vegetation type, occupying 33.7% of the study area. The average SMC depends on local topography [15, 16], so the sites were selected for similarity of gradient, aspect, shape, area, and elevation (Table 2), ensuring that the plant-soil interactions were the major factors influencing SMCs and soil properties.

**Table 2:** Characteristics of vegetation types.

Forest stands	Coordinates	Altitude (m)	Aspect	Slope (°)	Root depth (cm)	Average height (m)	DBH (cm)	Coverage (%)
AL	44°13′10.3″N, 118°44′40.6″E	1215	S	15.5	40–58	10	9–47	90
QM	44°13′13.8″N, 118°44′41.8″E	1212	S	16.2	36–45	3	5–20	85
UG	44°13′17.3″N, 118°44′38.9″E	1211	S	14.5	18–25	0.8	__	94

Notes: AL, artificial *Larix* spp.; QM, *Quercus mongolica*; UG, unused grassland.

### Rainfall data

Rainfall was measured with a tipping-bucket rain gauge (ONSET, USA) with a resolution of 0.2 mm, installed at the site of open grasslands. Rainfalls were recorded as separate events if the interval exceeded eight hours.

### Soil-moisture data

Soil moisture was recorded at 15 min intervals by ECH2O EC-TM soil-moisture sensors (Decagon Devices Inc. USA) with three replicates at the three vegetation sites during the principal soil-water recharge period. The sensors were installed at different root zones at depths of 10, 20, 30, and 40 cm for the three covers. Sensors were placed under three sample trees at 35 cm south of the tree for each forest site, and at three locations along the diagonal of the grassland site.

### Soil-property data

The soil properties for each vegetation site were analyzed for understanding their influence on SMC. Soil samples from the three were collected at depths of 10, 20, 30, and 40 cm, following the observation methodology for long-term forest ecosystem research (LY/T1952–2011). The samples were analyzed in the laboratory for pH, soil texture (USDA classification,US Department of agriculture 1956), bulk density, and soil organic matter and total nitrogen contents following the procedures of Hai[17]. These parameters were measured only once because they usually do not change measurably within short time periods.

### Data Analysis

No single location at a site represented soil moisture of a vegetation type or a soil layer, so we collected a large number of soil-moisture data from four soil depths at three locations every 15 min at each site. The data must be averaged to determine the temporal variation in soil moisture among the vegetation types and soil layers. Based on the equations proposed by Tyagi et al. [1] and Qiu et al. [18], we computed the mean monthly, daily and 15-min soil moistures in the soil layers and vegetation sites:

- Mean SMC of a site (M_i_)

$$M_{i}\;=\;\frac{1}{N_{l}N_{t}}\sum_{j\;=\;1}^{l}\sum_{k\;=\;1}^{t}M_{ijk}$$

- Mean SMC at a soil depth (M_j_)

$$M_{j}\;=\;\frac{1}{N_{p}N_{t}}\sum_{i\;=\;1}^{N_{p}}\sum_{k\;=\;1}^{N_{t}}M_{ijk}$$

- Time-averaged SMC (M_t_)

$${M_{t}\;=\;\frac{1}{N}\sum_{k\;=\;1}^{N_{t}}M}_{ijk}$$

- Mean maximum SMC for the four soil depths of a site (M_max_)

$$M_{max}\;=\;\frac{1}{N_{l}N_{t}}\sum_{i\;=\;1}^{N_{p}}\sum_{k\;=\;1}^{N_{t}}A_{ijk}$$

- Mean minimum SMC for the four soil depths of a site (M_min_)

$$M_{min}\;=\;\frac{1}{N_{l}N_{t}}\sum_{i\;=\;1}^{N_{p}}\sum_{k\;=\;1}^{N_{t}}I_{ijk}$$

where i is a site location, j is a soil depth, k is a sampling time, N_p_ is the number of sites, N_l_ is the number of sampling depths, N_t_ is the number of sampling times, A is the maximum SMC for the four depths, and I is the minimum SMC for the four depths.

The statistical analyses were performed using SPSS software and the figures and tables were generated using Microsoft Excel. The relationships between SMC and soil properties were analyzed by Pearson correlation analysis. The data set was analyzed for monthly and daily averages and for 15 min intervals based on similar measurement frequencies for precipitation and soil moisture.

## Results

### Variations in Soil Moisture with Depth for the Three Vegetation Types

The maximum, minimum, and mean SMCs, standard deviation (SD), and coefficient of variation (CV) at each soil depth under the three covers are shown in Table 3. The SMC throughout the 40-cm depth was generally higher for QM than for the other two covers. The maximum soil moisture was higher for QM (26.9%) and AL (23.4%) than for UG (22.7%). AL had the lowest M_min_ (10.5%) and M_i_ (14.5%) and the largest difference between M_max_ and M_min_ of the three covers.

**Table 3:** Soil-moisture content at each soil depth for the three vegetation types from June to September.

Land cover	Soil depth (cm)	M_max_ (%)	M_min_ (%)	M_i_ (%)	SD (%)	CV (%)
AL	10	30.2	12.6	17.9	3.8	21.0
	20	25.9	10.9	15.1	3.0	20.0
	30	21.3	9.3	13.6	2.8	20.5
	40	16.1	9.3	11.6	1.6	13.9
	Average	23.4	10.5	14.6	2.8	18.9
QM	10	30.4	16.4	22.5	2.8	12.5
	20	27.2	15.1	20.6	2.6	12.4
	30	24.9	16.3	21.4	2.7	12.5
	40	24.9	17.2	20.0	1.9	9.6
	Average	26.9	16.3	21.1	2.5	11.8
UG	10	25.8	8.8	16.1	3.0	18.7
	20	22.3	13.9	17.2	2.1	12.0
	30	22.5	12.2	16.6	3.1	18.7
	40	20.2	13.5	16.2	1.8	10.9
	Average	22.7	12.1	16.5	2.5	15.1

Notes: AL, artificial *Larix* spp.; QM, *Quercus mongolica*; UG, unused grassland; M_max_, mean maximum soil moisture for the four depths of the site; M_min_, mean maximum soil moisture for the four depths of the site; M_i_, mean soil moisture for the four depths of the site; SD, standard deviation; CV, coefficient of variation.

The coefficient of variation(CV)of SMC was higher in the surface layers than in the deeper layers for all three vegetation types. M_max_, M_min_, and M_i_ for AL had maximum attenuation with soil depth, and M_i_ for AL decreased from 17.9% in the top layer of soil to 11.6% at the 40 cm depth. AL also had the highest variation (CV 13.9–21.0%) in SMC from June to September relative to the other two covers. QM had the highest M_max_ and M_i_ for the top layer of soil and the smallest attenuation with regards to soil depth, particularly at the 30-cm and 40-cm depths. Soil moisture varied little from June to September for QM (CV 9.6–12.5%); the CVs among the first three depths were similar (CV 12.46±0.06%). M_max_ for UG generally decreased with depth, while the highest M_i_ was at a depth of 20 cm (17.2%), and the lowest M_i_ was in the top layer of soil due to the high evapotranspiration. SMCs for UG were moderately variable (CV 10.9–18.7%) from June to September.

### Monthly Variation of Soil-Moisture Content under Different Vegetation Types

**Fig 1:**
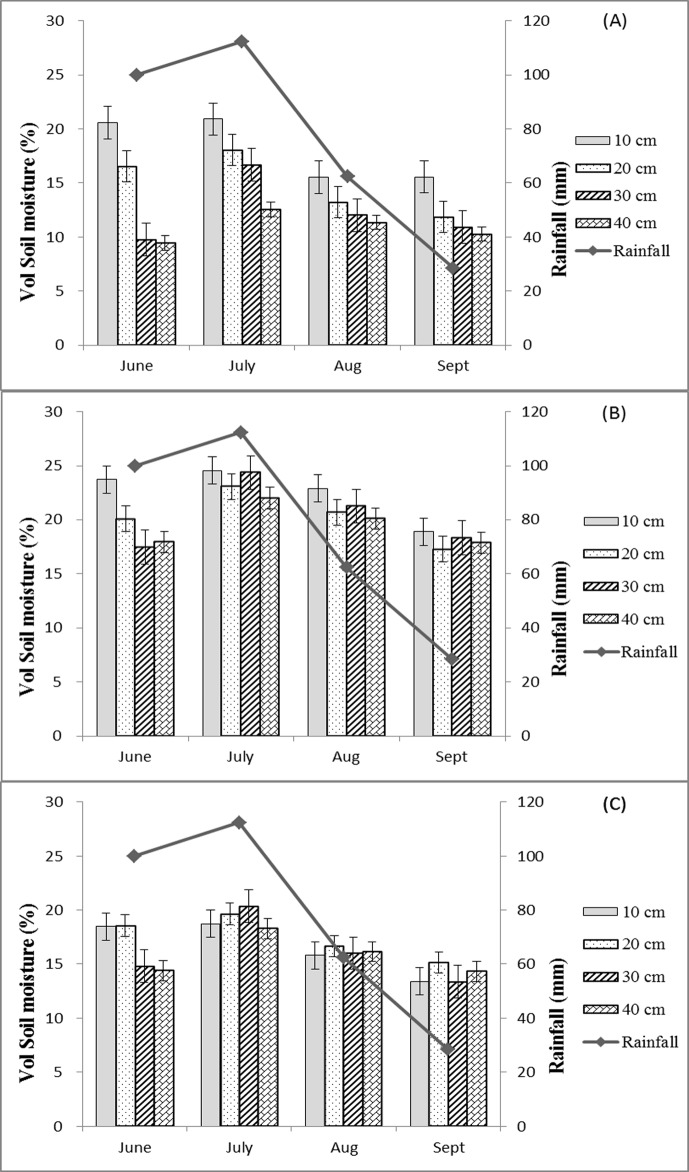
Monthly variation in soil moisture at the various depths for the three vegetation types during the rainy season. (A) Soil-moisture content of AL, (B) soil-moisture content of QM, and (C) soil-moisture content of UG. Notes: AL, artificial *Larix* spp.; QM, *Quercus mongolica*; UG, unused grassland.

Thirty-one rainfalls totaling 307 mm were recorded during the observation period. Continuous heavy rains fell at the end of June and July. The average SMCs at each soil depth for the three covers were positively correlated with the total rainfall for each month (Fig. 1). SMCs were relatively low in June, reached a peak in July, and then significantly decreased with the decrease in rainfall in August and September. The variation in mean SMC between each month was in the order of AL > UG > QM. The differences in SMC between each soil depth for the three covers were obvious in June and decreased most in August and September, especially for UG and QM.

### Daily Variation of Soil-Moisture Content under Different Vegetation Types

**Fig 2:**
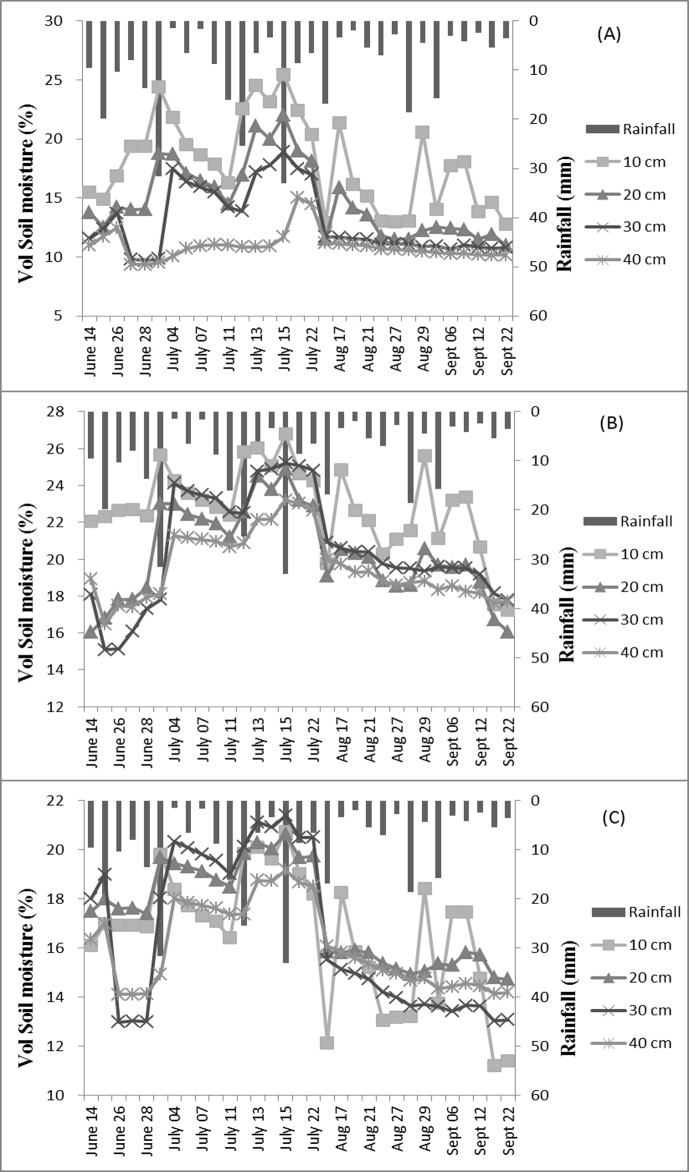
Daily variation of soil moisture at different depths for the three vegetation types. (A) Soil-moisture content of AL, (B) soil-moisture content of QM, and (C) soil-moisture content of UG. Notes: AL, artificial *Larix* spp.; QM, *Quercus mongolica*; UG, unused grassland.

Daily SMC was positively correlated with rainfall (*p* < 0.05) for the three covers. High SMCs for the three types of cover usually corresponded to heavy rains and decreased on sunny days due to strong evapotranspiration (Fig. 2). Daily average SMCs on rainy days at 30 cm and 40 cm depths for AL were relatively low and stable (10.15–11.71%), with the coefficient of variation of the SMC at the 40 cm depth changing from 10±1.8% to 15±1.5% only when the amount of rain reached 33 mm. Soil moisture on rainy days was higher for QM than the other two covers, with average moisture content at the various depths descending in the order 10 cm (22.82%) > 30 cm (20.31%) > 20 cm (20.58%) > 40 cm (19.59%). The variation in SMC at the 10-cm depth on rainy days was lower for QM than for the other two covers. Daily SMC of UG in the top layer (10 cm) was often the lowest of any soil depth due to high evapotranspiration and fluctuated greatly with rainfall due to the lack of canopy protection.

Initial levels of soil moisture played an important role in the changes in SMC. The resupply of soil water from rain was weakened with long intervals between rains. Very dry soil formed during the long interval (23 days) between the rainfall on July 22 and August 17. The 16.8 mm of rain that fell on August 17 failed to increase soil moisture adequately, and SMC was significantly lower than on any other rainy day for the three vegetation types (Fig. 2).

### Variation of Soil-Moisture Content at 15-Minute Intervals under Different Vegetation Types

Few rainfalls influenced the SMC at the 40-cm depth, so we chose to analyze the heaviest rainfall in each month that led to significant changes in SMC. The rainfalls on June 30 and July 15 had higher amounts and intensities, and the precipitation was concentrated over a short period of time. The rainfalls of August 28 and September 5 had lower amounts and intensities and spanned a long period with a brief pause. SMC was recorded every 15 min, and rainfall was recorded every hour.

**Fig 3:**
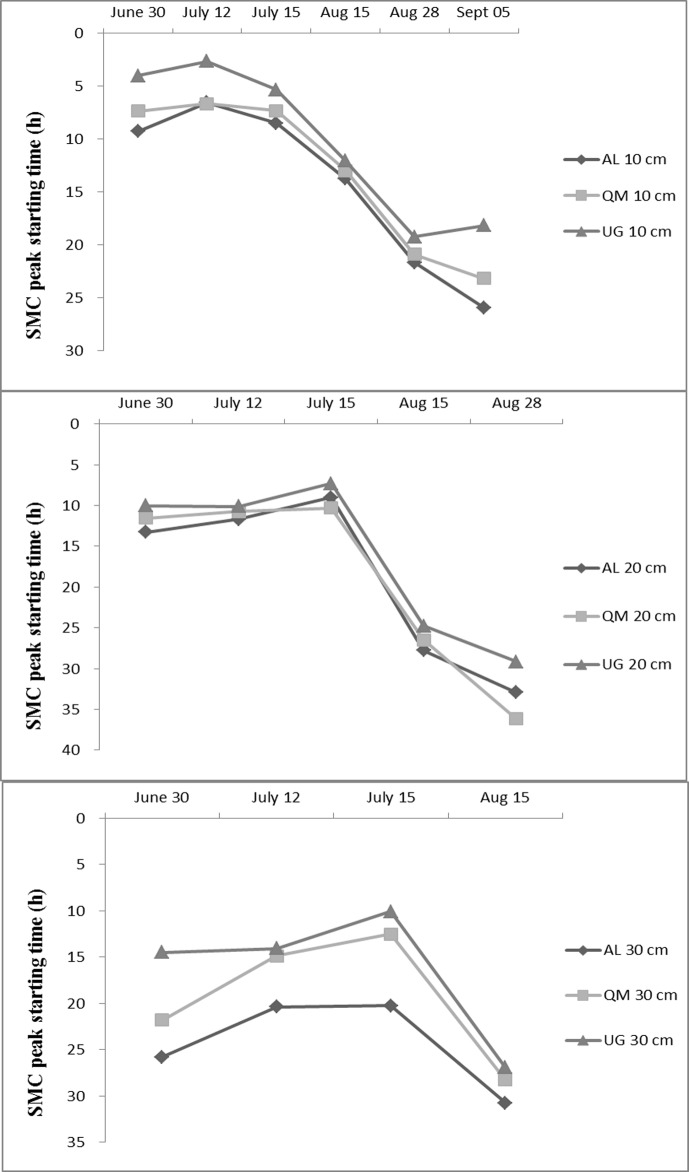
The onset of peak soil-moisture content (SMC) at each soil depth for the three vegetation types for individual rains. Notes: AL, artificial *Larix* spp.; QM, *Quercus mongolica*; UG, unused grassland.

Changes in SMC lagged following a rain. The time of onset of peak SMC at each soil depth for the three vegetation types is shown in Fig. 3. The order of onset of peak SMC at each depth was UG > QM > AL. The lag was longer as the amount and intensity of rain decreased. The shortest times to the onset of peak SMC for the six heaviest rainfalls were 2.63, 6.67, and 6.55 h in the top soil layer and 10.07, 12.50, and 20.23 h at the 30 cm depth for UG, QM, and AL, respectively. The slowest times to the onset of peak SMC at the 30 cm depth were 26.9, 28.25, and 30.75 h for UG, QM, and AL, respectively, after which the peak was maintained or repeated over a period of time, and then SMC gradually declined.

**Fig 4:**
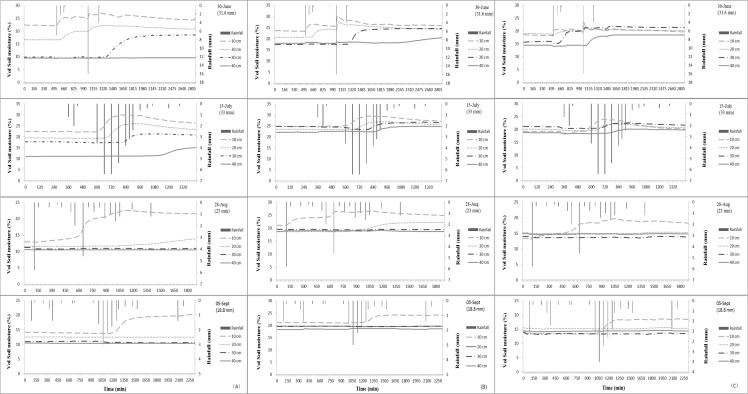
Fluctuation of soil-moisture content with rainfall for the three vegetation types for four single rains. (A) Soil-moisture content of artificial *Larix* spp., (B) soil-moisture content of *Quercus mongolica*, and (C) soil-moisture content of unused grassland.

The fluctuation in SMC for the three vegetation covers was consistent with the characteristics of the four rainfalls (Fig. 4). The amount and intensity of rain had more influence on soil moisture than duration. Surface soil was more sensitive to rain, especially for UG. Surface SMC for UG increased quickly when rainfall reached 3 mm, but surface SMC for AL and QM increased only when rainfall reached 5.5–6.5 mm due to canopy interception and the water-holding capacity of the litter. SMC of the 20–40 cm layer showed no obvious change when rainfall was less than 23 mm for all three covers, which indicated that the dry surface soil of UG absorbed more rainwater than the other two vegetation types.

### Soil Properties under Different Vegetation Types and Correlation with Soil Moisture

**Fig 5:**
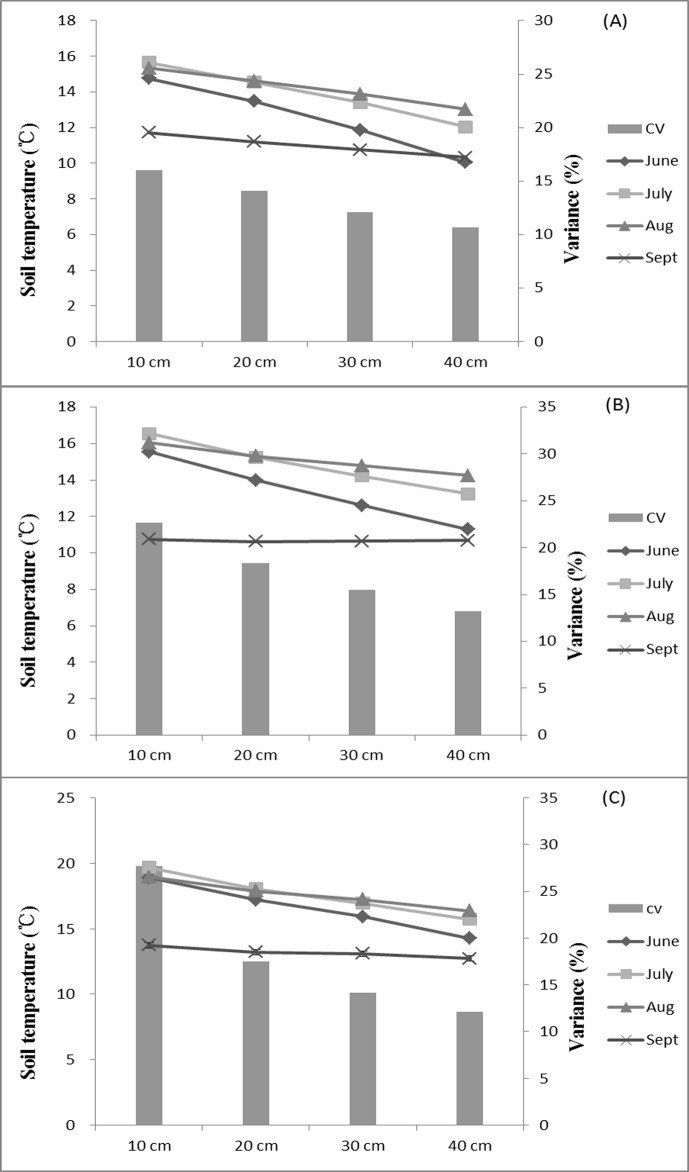
Soil temperatures for the three vegetation types during the rainy season. The variances (CVs, %) are also shown. (A) Soil-moisture content of artificial *Larix* spp., (B) soil-moisture content of *Quercus mongolica*, and (C) soil-moisture content of unused grassland.

Soil moisture is affected by soil temperature [19]. Mean temperature (0–40 cm) for the three covers during the period of observation was in the order AL (12.92°C) < QM (13.49°C) < UG (16.24°C), and the coefficient of variation of temperature was in the order AL (11.01–14.22%) < QM (10.68–15.10%) < UG (13.19–17.62%). Soil temperature and the variance in soil temperature both decreased as soil depth increased (Fig. 5). Soil temperature and moisture were negatively correlated in surface soil but were positively correlated in deeper soil (30-cm and 40-cm depths) (Table 4). This relationship was more significant for UG than for AL and QM and was more significant in August and September.

**Table 4:** Pearson correlations between soil temperature and soil moisture for the three vegetation types.

Month	AL				QM				UG			
	10 cm	20 cm	30 cm	40 cm	10 cm	20 cm	30 cm	40 cm	10 cm	20 cm	30 cm	40 cm
June	-0.10	0.25**	0.32**	0.17*	0.33**	0.28**	0.12*	0.28**	0.20**	0.27**	0.34**	0.38**
July	-0.43**	-0.37**	0.28**	0.77**	-0.05	-0.11*	0.18*	0.61**	-0.06	-0.06	0.30	0.11
Aug	-0.31**	-0.01	0.72**	0.77**	0.04	-0.30**	0.79**	0.79**	-0.33**	0.74	0.83**	0.91**
Sept	-0.55**	-0.63**	0.26**	0.93**	-0.32**	0.49**	0.63**	0.66**	-0.45**	0.86	0.91**	0.94**

Notes: AL, artificial *Larix* spp.; QM, *Quercus mongolica*; UG, unused grassland;

* *p* < 0.05;

** *p* < 0.01.

Soil properties are highly correlated with SMC [12, 14]. Average 0–40 cm soil bulk density was lower for AL and QM (both 1.04 g_/_cm^3^) than for UG (1.23 g_/_cm^3^). UG had relatively lower soil porosity and soil organic matter, total nitrogen, and total potassium contents than AL and QM (Table 5). The soil type was silty loam for all three vegetation types; silt constituted the largest proportion of the soil texture (50.20–58.80%), followed by sand (17.60–35.55%) and clay (10.40–13.60%). As shown in Table 6, the maximum SMC was positively correlated with soil porosity, soil organic matter and total nitrogen contents, and negatively correlated with pH and soil bulk density. Mean SMC, however, was significantly correlated only with pH and soil organic matter. The correlation between SMC and soil texture at the study area was not significant.

**Table 5:** Soil characteristics of the three vegetation types.

Vegetation type	Soil layer	pH	total nitrogen (g/kg)	total phosphorus (g/kg)	total potassium (g/kg)	soil organic matter (%)	soil bulk density (g/cm^3^)	soil porosity (%)	Sand (%)	Silt (%)	Clay (%)
AL	10 cm	5.60	3.01	1.17	29.32	63.69	0.88	63.25	20.60	53.10	12.70
	20 cm	5.95	2.75	1.16	29.83	58.25	0.92	59.65	35.55	51.88	12.57
	30 cm	5.88	2.53	1.25	29.43	53.40	1.05	56.68	20.70	51.20	12.00
	40 cm	5.90	2.32	1.07	29.27	48.49	1.32	46.73	20.90	50.20	12.90
QM	10 cm	5.21	3.15	1.24	29.68	69.97	0.97	62.99	20.20	54.40	12.00
	20 cm	5.37	2.73	1.10	29.10	60.08	0.97	59.22	33.01	53.67	13.32
	30 cm	5.58	2.67	1.08	28.93	57.60	1.03	58.21	19.40	53.40	12.60
	40 cm	5.75	2.26	1.04	28.85	48.48	1.20	49.88	17.60	57.80	10.40
UG	10 cm	5.81	2.80	1.03	29.95	55.79	1.09	55.59	18.40	58.80	12.30
	20 cm	5.71	2.58	0.92	29.32	51.45	1.15	55.03	29.56	57.08	13.36
	30 cm	5.70	2.37	0.88	29.25	48.01	1.23	54.11	19.50	56.30	13.60
	40 cm	5.73	1.76	0.86	29.15	33.51	1.45	48.16	18.10	50.90	13.10

Notes: AL, artificial *Larix* spp.; QM, *Quercus mongolica*; UG, unused grassland.

**Table 6:** Correlations among soil properties and mean soil-moisture contents.

	pH	total nitrogen	total phosphorus	total potassium	organic matter	bulk density	soil porosity	sand	silt	clay	M_max_	M_min_
total nitrogen	–0.47											
total phosphorus	-0.21	0.69*										
total potassium	0.16	0.49	0.32									
organic matter	-0.55	0.98**	0.76**	0.38								
bulk density	0.35	-0.90**	-0.75**	-0.35	-0.91**							
soil porosity	-0.54	0.89**	0.63*	0.37	0.88**	-0.93**						
Sand	0.01	0.27	0.15	0.23	0.27	-0.44	0.34					
Silt	-0.15	0.21	-0.31	0.08	0.13	-0.07	0.07	-0.14				
Clay	-0.07	-0.07	-0.42	0.07	-0.14	-0.12	0.05	0.36	-0.25			
M_max_	-0.64*	0.77**	0.47	0.24	0.78**	-0.80**	0.86**	-0.15	0.34	-0.3		
M_min_	-0.69*	0.06	-0.06	0.59*	0.17	-0.10	0.50	-0.04	0.25	-0.26	0.44	
M_i_	-0.84**	0.40	0.12	0.29	0.58*	-0.39	0.18	-0.03	0.40	-0.23	0.73**	0.89**

Notes: M_max_, mean maximum soil moisture for the four depths of the site; M_min_, mean maximum soil moisture for the four depths of the site; M_i_, mean soil moisture for the four depths of the site.

* *p* < 0.05,

** *p* < 0.01.

## Discussion

### Effect of Vegetation Type on Soil Moisture

Vegetation cover directly affects the soil-moisture regime and thus the hydrological behavior of a watershed. SMC can be higher for forests than for grassland, but not always [20–22]. Peng [23] reported that the average annual SMC in the Heihe River basin is about 50% higher in spruce forest than in grassland. Bharati [20] reported that SMC in a riparian buffer in the US is in the order of switch grass > grass filter > silver maples > pasture. Garcia- Estringana [5] indicated that soil moisture to a depth of 80 cm is generally lower under forest cover than under grasses on the southern margin of the Pyrenees. Previous studies have also shown that SMC in forests is positively correlated with tree density [12, 21, 24]. Tyagi [1] reported that soil moisture in the lower Himalayan region of India decreases with canopy density, and Duan [12] reported that locations with denser vegetation covers tend to have higher soil moistures.

The relationship between tree density and SMC in this study is not in complete agreement with previous studies, indicating that the comparison of soil moisture between forest and grassland is dependent on tree species. In this study, the small QM trees had the highest SMC (M_max_ and M_i_), followed by grassland (M_i_) and the tall AL trees (M_i_). Mean SMC was lower for AL than for QM, despite the higher canopy density of AL, perhaps because the high AL canopy likely intercepted a large proportion of the rainwater in this semiarid region and because the tall trees would transpire large amounts of soil water in summer.

### Variation of Soil Moisture with Soil Depth

Moisture content tends to be relatively complex and variable due to the high recharge of soil water from precipitation and evapotranspiration [15, 25]. A high variance in soil moisture has been associated with wet periods [26], but Charpentier and Groffman [27] found no significant relationship between variance and mean moisture content. Our results support those by Charpentier and Groffman: the relationship between variance and mean SMC was not significant for the three vegetation types (*p* = 0.12).

Soil moisture fluctuates with rainwater rechargeable depth but is stable below the rechargeable depths [23, 28]. Vegetation reduces the amount of water that reaches the ground by intercepting rain and reducing the amount of soil water lost to evapotranspiration and uptake by roots [5]. In this study, most rainfalls poorly recharged the soil below 40 cm due to low rainfalls, canopy interception, and the high water-holding capacities of the litter and surface soil. The rainfall threshold for increasing surface SMC was in the order of AL > QM > UG. The patterns of soil moisture vary with depth. Deep-rooted trees normally transpire more water and can extract water from deeper soil than can many shallow-rooted grasses [22, 28]. Our results show that soil moisture decreased with depth in the forest sites, whereas it increased within the upper 30 cm and then decreased below 30 cm in the grassland. More water was consumed from the deeper soil by the deep-rooted trees, more water was consumed from the surface soil by the shallow-rooted (18–25 cm) grass; and soil water was recharged less in deep (40 cm) soil in both forest and grassland.

Our results are consistent with those by Garcia-Estringana [5], who reported that an increase in soil moisture was delayed more in a forest than in grassland. The dense canopies and dry deep soil layers of the AL and QM forests led to the slowest times to the onset of peak SMC at a depth of 30 cm. Intensive monitoring at 15 min intervals identified some further features of soil moisture. Rain falls through canopies and moves within the soil as a medium, and different plant-soil interactions have different impacts on changes of SMC. The transfer of rainwater to deeper soil layers can be relatively slow. The slowest times to the onset of peak SMC at a depth of 30 cm for all rains in 2013 were 26.9, 28.25, and 30.75 h for UG, QM, and AL, respectively. With an increase in water supply, the peak SMC was maintained or repeated over a period of time, and then gradually declined after the rain stopped. The process of rainwater transfer, which may require 1–2 days, was highly dependent on the characteristics of the rain, vegetation structure, and soil properties. First, unlike in double-ring infiltration experiments, natural rainwater recharging is a process of intermittent accumulation, so soil infiltration would vary with the amount and intensity of rain and with the interval between rains. Second, rainwater is intercepted by canopies, runs down tree trunks, is held in the litter, and is then transferred to the soil layers; and the water can still enter the soil due to gravity when the rain stops. Third, water is adsorbed by surface soil during the wetting phase by molecular forces in the form of thin films, and as rainwater recharge increases, water fills the pores in the soil due to capillary force and gravity. Water will continue to infiltrate to deeper soil only when the surface soil is saturated.

### Effect of Soil Temperature and Other Properties on SMC

An increase in the temperature of surface soil corresponds to a decrease in the SMC [29], and soil-water content correlates negatively with diurnal temperature changes [30, 31]. We further found that temperature and moisture in the surface soil (10 cm) are generally negatively correlated, but the variations between temperature and moisture are positively correlated in deeper soil (30 cm and 40 cm depths). Evaporation from the surface soil is high when the soil temperature is high. The specific heat and the conduction of heat has been reported to be about 3 and 24 times higher, respectively, in soil than in air [30], indicating that soil with higher humidity is better able to conduct thermal energy from the surface soil during the day and maintain a stable SMC at night, which may account for the positive relationship between soil temperature and deep SMC in this study.

Soil properties have an important influence on soil moisture [32]. SMC was positively correlated (*R* ^2^ > 0.7) with silt-clay, organic-matter, total nitrogen, and total phosphorus contents in Horqin in Inner Mongolia [12]. In this study, the maximum SMC was significantly correlated with soil porosity, pH, soil bulk density, and soil organic matter and total nitrogen contents, but mean SMC only correlated with pH and soil organic matter content. This is likely because maximum SMC represents the capacity of soil to hold water under conditions of abundant rainfall, which correlates highly with soil properties, but the mean SMC between June and September was influenced by many other factors (e.g. precipitation, evapotranspiration, runoff, infiltration, and canopy cover). Soil organic matter content plays a key role in maintaining soil structure and nutrient and moisture levels [32]. In this study, soil pH and soil bulk density are negatively correlated with SMC and soil organic matter content. Soil acidity is mainly due to humus and organic matter, and porous soil has a low soil bulk density, indicating that soils with low pH and soil bulk density have high SMCs and soil organic matter content.

## Conclusions

Soil moisture content was monitored and compared for three types of vegetation in the Saihanwula National Nature Reserve in Inner Mongolia, China. Based on mean soil moisture content and soil moisture variability, *Quercus mongolica* is best able to conserve soil-moisture resources, followed by grassland and *Larix spp*. The study also illustrated the soil moisture dynamics for the three vegetation types at different soil depths. Soil moisture content and its variability decreased with soil depth, and soil moisture to a depth of 40 cm was poorly recharged by rainwater for the three vegetation types. Temporal changes in soil moisture correlated with rainfall, and the speed of onset of peak SMC at each depth for the vegetative covers was in the order of grassland > *Quercus mongolica* > *Larix spp*. Many soil properties such as temperature, pH, soil organic matter content, and soil bulk density affected the SMC. *Quercus mongolica* had higher contents of nutrients and a lower soil bulk density, which likely increased the ability of the soil to hold water.

This study has provided support for the management of soil-water conservation and hydrological processes in arid and semiarid areas. Further study of forest structure and age may provide more effective strategies.
